# High-Throughput Screen Identifying the Thiosemicarbazone NSC319726 Compound as a Potent Antimicrobial Lead Against Resistant Strains of *Escherichia coli*

**DOI:** 10.3390/biom8040166

**Published:** 2018-12-07

**Authors:** Carmen Sadaka, Peter Damborg, Jeffrey L. Watts

**Affiliations:** 1 Department of Veterinary and Animal Sciences, University of Copenhagen, Stigboejlen 4, 1870 Frederiksberg C, Denmark; 2 Zoetis Global Therapeutics Research, 333 Portage Street, Kalamazoo, MI 49007, USA; pedam@sund.ku.dk

**Keywords:** sulfonamide resistance, antifolate, *Escherichia coli*, antimicrobial lead, drug discovery, multidrug resistance

## Abstract

Antibiotic discovery is vital when considering the increasing antimicrobial resistance threat. The aim of this work was to provide a high-throughput screen (HTS) assay using multidrug-resistant *Escherichia coli* strains to enable further research into antimicrobial lead discovery and identify novel antimicrobials. This study describes a primary HTS of a diverse library of 7884 small molecules against a susceptible *E. coli* strain. A secondary screening of 112 molecules against four *E. coli* strains with different susceptibility profiles revealed NSC319726 as a potential antimicrobial lead serving as a novel template. NSC319726 is a good candidate for an analoguing program.

## 1. Introduction

Recently, multidrug-resistant bacteria have emerged, threatening human health at a global level [1]. Considering this threat, it is vital to identify novel antimicrobial classes [2,3,4]. Nonetheless, investments in antimicrobial lead discovery [5] and the number of approved novel antimicrobials are on the decline [3,4,6,7]. Despite discovery efforts towards mechanistic-based high-throughput screening (HTS) [8], the pharmaceutical industry has mostly abandoned the anti-infective arena because of its limited financial attractiveness, the scientific challenges inherent to antibiotic drug discovery, and the complex regulatory framework [5]. A number of measures (collaborative programs, new funds, regulatory measures, etc.) have been launched in the past years aiming to revive the antimicrobial pipeline and to overcome bottlenecks in the development of new antibiotics. However, these initiatives still fall short of providing all the necessary tools to cope with the magnitude of the public health challenges faced today. Filling the antimicrobial drug discovery pipeline has never been as challenging as now, with the currently used scaffolds and protein targets being overexploited. The identification of a novel anti-infective lead can be achieved by revitalizing old antimicrobial targets with new inhibitors or by identifying new antimicrobial targets. New inhibitors can be molecularly tailored old scaffolds, new scaffolds, or repurposed compounds. Folate biosynthesis is a well-established antimicrobial target validated by the use of clinically effective sulfonamides and trimethoprim (TMP). The ongoing effort to identify novel antifolates is stimulated by the central biological importance of folates and the absence of its biosynthetic pathway in eukaryotes [9]. Previous antifolate discovery efforts have focused on the folic acid pathway as the primary metabolism to folates, specifically on the dihydropteroate synthase and the dihydrofolate reductase enzymes within the folic acid pathway. However, unexplored molecular targets remain in earlier steps of the folate synthesis, such as within the chorismate and shikimate pathways. In an attempt to identify novel antifolate leads effective against multidrug-resistant strains, a novel whole cell HTS assay specific to the chorismate pathway was developed.

## 2. Materials and Methods

### 2.1. Bacterial Isolates

Three *Escherichia coli* American Type Culture Collection (ATCC) strains 25922, 86980, and 29181 were used in this study along with the clinical isolate *E. coli* AHDRCC 81113. In order to identify antifolate leads effective against resistant strains, the tested strains used in the assay had different susceptibly profiles towards sulfamethoxazole (SMX), trimethoprim/sulfamethoxazole (SXT), and TMP (Sigma-Aldrich, St. Louis, MO, USA). The tested resistant strains were also resistant to antimicrobials other than antifolates (including ampicillin, florfenicol, kanamycin, penicillin, streptomycin, and tilmicosin). *Escherichia coli* ATCC 25922 was susceptible to SMX, SXT (concentration ratio 1:19), and TMP and was a Clinical and Laboratory Standards Institute (CLSI)-recommended quality control strain for susceptibility testing of potentiated sulfonamides and other antimicrobials [10,11]. *E. coli* ATCC 86980 contained the pLS88 plasmid conferring resistance to kanamycin, streptomycin, and sulfonamide. *E. coli* ATCC 29181 was an ATCC strain resistant to streptomycin and TMP. *E. coli* AHDRCC 81113 was a clinical isolate from the Zoetis culture collection (*E. coli* AHDRCC 81113, collected in 2014 from cattle from Ontario, Canada) that is resistant to ampicillin, florfenicol, penicillin, SMX, SXT (1:19), tilmicosin, and TMP. Susceptibility profiles to NSC319726 (Cayman Chemical, Ann Arbor, MI, USA), SMX, SXT (1:19) and TMP were confirmed with minimum inhibitory concentration (MIC) testing (*n* = 2) by the broth microdilution procedure as described by the CLSI document Vet01-A4 [10]. Quality control was conducted using CLSI-recommended quality control strains and antimicrobials. Quality control strains used were *Enterococcus faecalis* ATCC 29212, *Staphylococcus aureus* ATCC 29213, and *E. coli* ATCC 25922. Quality control antimicrobials used were ampicillin, amoxicillin/clavulanic acid combination (2:1, v:v), and SXT combination (1:19) (Sigma-Aldrich) [10,11].

Following the propagation procedure indicated by the manufacturer (ATCC, Manassas, VA, USA), all strains were frozen in trypticase soy broth containing 10% glycerol and maintained at −80 °C until revived and sub-cultured for testing. Inoculated plates were incubated for 18 to 24 h at 35 °C ± 1 °C in 5% ± 2% CO_2_ and sub-cultured prior to use. The inoculum used in the primary and secondary screen was prepared via the direct colony suspension method as described in the CLSI document Vet 01-A4 [10] and adjusted to 10^5^ colony-forming units (CFU)/mL in cation-adjusted Mueller Hinton broth (CA-MHB) supplemented with thymidine phosphorylase (TP) (200U/L).

The CA-MHB (Thermo Fisher Scientific, Waltham, MA, USA) was prepared as per the instructions of the manufacturer and adjusted to pH 7.3 ± 0.1 prior to autoclaving [10]. After autoclaving, CA-MHB was stored overnight at room temperature. TP (Becton, Dickinson and Company, Franklin Lakes, NJ, USA) (200U/L) was added to CA-MHB prior to use in the HTS assay.

### 2.2. High-Throughput Screens

Bacterial growth inhibition was monitored by using the BacTiter-Glo™ microbial cell viability (Thermo Fisher Scientific) fluorescence-based assay to quantify adenosine triphosphate content in viable cells. Automation for high-throughput screening included assay reagent handling in 384-well format (Corning 3571; black flat bottom polystyrene tissue culture-treated sterile microplates), compound addition (Multivolume ATS Acoustic Bravo Liquid Dispenser, Atlantic Lab Equipment, Salem, MA, USA), inoculated media and BactTiter Glo addition (Multidrop Combi Reagent Dispenser, Thermo Fisher Scientific), and assay monitoring (Envision luminometer, Perkin Elmer, Waltham, MA, USA).

All robotic liquid handling, compound transfer, and plate reading were performed at Zoetis Global Therapeutics Research (Kalamazoo, MI, USA). Positive (high-percent effect (HPE)) (*n* = 16) and negative control (zero-percent effect (ZPE)) (*n* = 16) wells were added to each tested plate. High-percent effect controls consisted of 100 μg/mL of SXT (1:19 ) in dimethyl sulfoxide (DMSO), and ZPE controls consisted of DMSO only. Test compounds (0.3 μL of either compound, HPE or ZPE) were first added to each well in the plates followed by the inoculated media (30 µL containing 10^5^ CFU/mL). After 16 to 20 h of incubation at 35 °C ± 1 °C in 5% ± 2% CO_2_, the BactiterGlo reagent (30 µL) was added. Plates were covered with a low-evaporation lid seal, and contents were briefly mixed (2 min) on an orbital shaker (Standard Analog 1000 Orbital Shaker, Troemner™, Thermo Fisher Scientific) to induce cell lysis. Fluorescence (Envision luminometer, Perkin Elmer) was read after 20–40 min of incubation at room temperature. 

In the primary screen, a library of 7884 compounds was screened in a 384-well plate setting against *E. coli* ATCC 25922. The library of compounds included a collection of Food and Drug Administration (FDA)-approved molecules, as well as synthetic small molecules, natural products, known bioactives, peptides, peptidomimetics, inorganic and metal-containing products sourced from Zoetis Global Therapeutics Research. Test compounds from the screening library were added to the reaction at a final concentration of 10 μg/mL in 0.99% DMSO. The secondary screen consisted of an 11-point titration (3.16 × dilution) of inhibitory compounds from the primary screen. Tested concentrations were 100, 31.65, 10.02, 3.17, 1, 0.32, 0.1, 0.03, 0.01, 0.003, and 0.001 µg/mL. A concentration of 100 µg/mL of SXT was used as both HPE and as a positive control. Two different titrations for SXT were tested on each run (*n* = 3) of the secondary screen. The primary screen was run in duplicates (N1 and N2), and the secondary screen was run in triplicates (N1, N2 and N3) on different days.

The PubChem Sketcher V2.4 online software (PubChem, National Center of Biotechnology Information, Bethesda, MD, USA) was used to draw the NSC319726 lead structure. Microsoft excel (Microsoft corporation, Redmond, Washington, USA), Tibco Spotfire (Tibco software Inc., Palo Alto, CA, USA), and GraphPad Prism v. 6.0 (GraphPad Software, La Jolla, CA, USA) software were used for data analysis. Briefly, dose–response curves and calculation of half maximal inhibitory concentration (IC_50_) (µg/mL) and pIC_50_ data (N1, N2 and N3) were made using Excel tools and GraphPad Prism software, v. 6.0. Briefly, individual concentration–effect curves were generated in GraphPad Prism by plotting the logarithm of the tested concentration for each of the different tested compounds (X) versus the corresponding percent inhibition values (Y) using least square (ordinary) fit. GraphPad Prism reports both the best fit IC_50_ and its log. The best fit IC_50_ values were calculated in GraphPad Prism using log (inhibitor) versus response-variable slope (four parameters) equation, where Y = Bottom + (Top − Bottom)/(1 + 10^((LogIC50 − X)∗HillSlope))^. IC_50_ data were converted to M from µg/mL in Excel as follows: IC_50_ (M) = IC_50_ (µg/mL)/(1000 ∗ MW), where MW is the molecular weight expressed in g/mol. pIC_50_ were calculated in excel as follows: pIC_50_ = −log [IC_50_ (M)]. Bland–Altman plots were drawn using Excel and Tibco Spotfire software. 

## 3. Results

The strain *E. coli* ATCC 25922 used in the primary screen was susceptible to SMX, SXT (1:19), and TMP (Table 1) [10,11]. Data of duplicate primary screens run on separate days (N1 and N2) were of high quality, with *Z*-prime statistical scores of 0.65 (N1) and 0.61 (N2), both > 0.5, indicative of an excellent assay, exhibiting a large separation band between HPE and ZPE (fold window = ZPE/HPE ~ 13 and ~ 32 for N1 and N2, respectively), good signal-to-noise ratios, and good signal-to-background ratios in the compound and control wells [12]. This was consistent with signal window (SW) values (6.11 (N1) and 4.91 (N2)) that were large enough and >2, indicative of a recommended assay [13]. All data were reported as percent effect of inhibition relative to the average of the ZPE and HPE (percent effect of inhibition = (sample luminescence−average ZPE)/(average HPE−average ZPE) ∗ 100)). Active molecules were identified as those showing more than 50% percent effect of inhibition. Using this threshold, 112 compounds were found to inhibit the growth of *E. coli* ATCC 25922, giving a primary hit rate of 1.42% over the entire screen. 

In the secondary screen, IC_50_ determinations were performed for the 112 active compounds from the primary screen. The secondary screen was run against each of *E. coli* ATCC 25922, *E. coli* ATCC 86980, *E. coli* ATCC 29181, and *E. coli* AHDRCC 81113, separately. Similarly to the primary screen, *Z*-prime statistical scores were > 0.5 (0.67 for N1, 0.72 for N2, and 0.68 for N3), and SW values > 2 (7.21 for N1, 8.46 for N2 and 7.51 for N3) [12,13]. 

Generally, a good agreement in Bland–Altman plots was observed in the IC_50_ values obtained from the three different secondary assay runs (N1, N2, and N3) for each strain (Figure S1, supplemental data) [14,15,16,17,18]. The Bland–Altman plot shows that the average differences in pIC_50_ are zero or very close to zero, indicating no significant bias between replicate assays (N1, N2, and N3) in each of the different tested strain [14,15,16,17,18]. Moreover, there were no trends in the data, and variability was reasonably uniform across the range of potency (artifacts, temporal and spatial plate effect) [14,15,16,17,18]. The limits of agreement (LoA) and minimum significant difference (MSD) were acceptable. Any positive outside the 95% confidence interval of the LoA and/or MSD interval was considered an outlier and was excluded from data analysis. The number of those outliers was very low (1.34%). Most outliers were seen among resistant strains (marked with numbers in Figure S1, supplemental data) and corresponded to nuisance hits.

**Figure 1:**
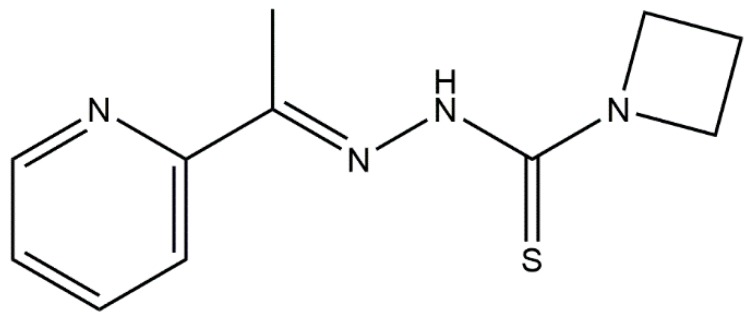
The chemical structure of the thiosemicarbazone NSC319726 lead.

In HTS lead discovery, pIC_50_ ≥ 6 corresponds to a positive and a potent inhibitor. However, in antimicrobial lead discovery, pIC_50_ ≥ 4 corresponds to a positive. The lower bound threshold at 95% CI of pIC_50_ calculated on the basis of data of the two different titrations of the positive control (SXT) (*n* = 6) was used as cut-off to determine hits during data analysis (pIC_50_ ≥ 4.26). For compounds where 4 < pIC_50_ < 4.26, pIC_50_ (N1, N2, and N3), values were averaged, and compounds were considered as hits if the average pIC_50_ ≥ 4.26 (Table 1). Additional criteria such as the overall appearance of the curve, <60% effect, high IC_50_, toxicity and safety issues were considered in the selection of hits. On the basis of these criteria, NSC319726 (Figure 1) was identified as a potential antimicrobial lead, effective against the tested susceptible and multidrug-resistant *E. coli* strains (IC_50_ averages ranging from 0.0021 to 0.0232 µM among the tested strains) (Table 2). 

Susceptibility results (*n* = 2) by the broth microdilution procedure [10] for NSC319726 with *E. coli* strains susceptible (*E. coli* ATCC 25922) and resistant (*E. coli* AHDRCC 81113) to antifolates showed that NSC319726 inhibited the growth of both antifolate-susceptible and -resistant strains (Table 3). This finding was consistent with the generated IC_50_ data (Table 2). The NSC319726 exhibited moderate MICs (128 μg/mL ± two-fold dilution) against both *E. coli* ATCC 25922 and *E. coli* AHDRCC 81113. The potency of NSC319726 was comparable in both antifolate-susceptible and -resistant *E. coli* strains tested. The potency of NSC319726 was also comparable to that of SMX alone against the antifolate-susceptible strain (*E. coli* ATCC 25922), with the consideration of one two-fold dilution difference in potency between SMX and NSC319726. Notably, *E. coli* AHDRCC 81113 is multidrug-resistant as it is resistant to antifolates (SMX, TMP, and SXT) and to ampicillin, florfenicol, penicillin, and tilmicosin. While SMX alone is ineffective against *E. coli* AHDRCC 81113, NSC319726 inhibited its growth with a MIC of 128 μg/mL (Table 3).

## 4. Discussion

In our search for novel antifolates, NSC319726 exhibited antimicrobial activity against the susceptible and multidrug-resistant *E. coli* strains tested (resistance to ampicillin, florfenicol, kanamycin, penicillin, streptomycin, SMZ, SXT, tilmicosin, and TMP) (Table 2). Considering the HTS data, the thiosemicarbazone NSC319726 was a potent inhibitor of all the tested strains (pIC_50_ values ≥ 4.26) (Table 2). Considering the MIC data, NSC319726 demonstrated adequate activity against the tested strains (MIC = 128 μg/mL ± two-fold dilution), warranting further investigation. NSC319726 inhibited the growth of multidrug-resistant *E. coli* strains and is highlighted as a novel template serving as a potential antimicrobial lead. 

Previously, NSC319726 was reported to inhibit the growth of mammalian cancer cell lines with a p53 mutation and to inhibit the growth of pathogenic fungi [19]. Here, we report for the first time NSC319726 as a potent inhibitor against multidrug-resistant *E. coli* strains (Table 2). The antifungal effect of NSC319726 against *Candida albicans* SC5314 is due to downregulation of ribosome biogenesis and protein synthesis [20]. On the basis of the antifungal data, we assume that the antimicrobial activity of NSC319726 is likely due to inhibition of protein synthesis through an interaction with the bacterial ribosome. On the basis of pIC_50_ values (≥4.26), NSC319726 is a more potent inhibitor than SXT in all the tested *E. coli* strains (Table 2). It is more potent against antifolate-susceptible *E. coli* ATCC 25922 (IC_50_ = 0.0033 µM) and SMX-resistant *E. coli* ATCC 86980 (IC_50_ = 0.0021 µM) than against *E. coli* ATCC 29181 resistant to TMP (IC_50_ = 0.0131 µM) and *E. coli* AHDRCC 81113 resistant to TMP, SMX, and SXT (IC_50_ = 0.0232 µM) (Table 2). Overall, the IC_50_ values (Table 2) indicate that the antimicrobial activity of NSC319726 against *E. coli* is similar to that previously observed against pathogenic fungi, including *Candida* species, *Aspergillus fumigatus*, and *Cryptococcus neoformans* [20]. Considering that the highest growth inhibition IC_50_ value obtained with NSC319726 against multidrug-resistant *E. coli* does not exceed by more than 2.7 times the highest growth inhibition IC_50_ value obtained with NSC319726 against pathogenic fungi, the cytotoxic IC_50_/growth inhibition IC_50_ data can be extrapolated (by dividing by 2.7, corresponding to the highest fold difference in IC_50_ growth inhibition between the two studies [20]) from the study conducted by Sun et al. [20] and is ~296-fold higher following a 24 h incubation of cell lines with NSC319726 and ~13- and 122-fold higher at 48 and 72 h (The toxicity of NSC319726 to human liver cell lines was expressed as ratios of IC_50_/MIC_50_ by Sun et al. Depending upon the time of incubation with the compound, their IC_50_ concentrations were 35-, 330-, and 825-fold higher to achieve inhibition than the concentration to achieve an MIC_50_, 72, 48, and 24 h post-treatment, respectively [20]). Despite this drop in fold difference, it remains considerably high, indicating limited toxicity at the tested concentrations. Since previously published cytotoxicity data indicate no significant toxicity of the compound to human and rodent cell lines, [20] NSC319726 constitutes an attractive lead compound with strong antimicrobial activity against multidrug-resistant *E. coli*. It is a good candidate for analoguing programs aiming to enhance its antimicrobial activity and reduce its cytotoxicity.

The challenge presented by the NSC319726 lead is the lack of any pre-existing information about its characteristics and its biological behaviour. Nonetheless, this hurdle can be easily overcome through the synthesis and evaluation of NSC319726 analogues. The synthesis of analogues will help understand the precise mechanism of action of the lead pharmacophore, define structure–activity relationship requirements, and reveal the impact of the various structural features on the antibacterial activity, toxicity, physicochemical properties, and pharmacokinetic parameters. The recorded MIC of NSC319726 against resistant strains (128 μg/mL) is the most crucial requirement used to guide such analoguing programs and structure activity relationship (SAR) studies. The SAR efforts would then have clearly defined biological goals including in vitro and in vivo activity, toxicity, as well as physicochemical properties and pharmacokinetic parameters. An NSC319726 analogue which succeeds in meeting the in vitro and then the in vivo criteria, passes toxicological profiling, and meets the PK requirements would then move forward the antimicrobial discovery pipeline, become a clinical drug candidate suitable for additional preclinical development, and ultimately enter Phase I human clinical trials. This is not an uncommon practice in drug discovery. In fact, linezolid, the first clinically useful oxazolidinone, portrays a good example of a successful antimicrobial that emerged from an analoguing program of the novel oxazolidinone antimicrobial class that had unknown characteristics and biological behaviour at the time [21]. The synthesis of NSC319726 analogues is straightforward and could be initiated from ketone and hydrazine. Chemical modifications will probe a number of structural features of the NSC319726 lead compound to assess their impact on antibacterial activity and toxicity. Chemical modifications can include the replacement of the pyridine with different heterocycles, the addition/modification of different alkyl chains and aromatic groups, the replacement of sulfur with oxygenation, the addition of other nitrogen ring systems, the addition of positive charges, and the addition of negative charges. After understanding the SAR structural constraints and mechanism of action, unnecessary structures can be eliminated. Following the confirmation of in vitro activity, should in vivo issues such as toxicity and physicochemical issues such as solubility arise, a prodrug structure can be synthesized. Finally, since peptides are easier to synthesize, are presented with better clearance, and have a less toxic profile than small organic compounds, the new lead structure can be converted into a non-standard dipeptide. The advantage of attempting to synthesize a non-standard dipeptide structure over a traditional dipeptide structure is the possibility of a longer half-life, which may further decrease the administered dose and toxicity.

## 5. Conclusions

The NSC319726 compound constitutes a potential antimicrobial lead serving as a novel template. NSC319726 exhibits a good antimicrobial activity against *E. coli*, irrespective of concomitant resistance to antifolates and to other antimicrobial classes. The thiosemicarbazone NSC319726 is suitable for a medicinal chemistry program aiming to develop novel analogues with enhanced antimicrobial activity and reduced toxicity. More in vitro and in vivo efficacy, toxicological, and pharmacokinetic studies are required to deem if NSC319726 pharmacophore will continue to serve as a lead antimicrobial and move forward as a pre-clinical candidate.

## Figures and Tables

**Table 1:** IC_50_ (µg/mL) and pIC_50_ data (N1, N2, and N3) for two titrations of SXT (SXT_1 and SXT_2) on each run (N1, N2, and N3) of the secondary screen and their corresponding average, standard deviation, sample size, confidence coefficient for 95%CI, margin of error, CI upper bound, CI lower bound, maximum (sample), minimum (sample), and range (sample and CI).

*Escherichia coli* ATCC 25922		
SXT (MW 633.68 g/mol)	IC_50_ (µM)	pIC_50_
SXT_1_N1	0.042	4.38
SXT_1_N2	0.017	4.773
SXT_1_N3	0.016	4.807
SXT_2_N1	3.29 × 10^−6^	8.483
SXT_2_N2	0.014	4.84
SXT_2_N3	0.003	5.587
Average	0.015	5.478
Standard deviation		1.523
Sample size		6
Confidence coefficient, for 95% CI		1.96
Margin of error		1.219
CI Upper bound		6.697
CI Lower bound		4.26
Maximum (sample)		8.483
Minimum (sample)		4.38
Range (sample)		4.103
Range (CI)		2.438

SXT: trimethoprim/sulfamethoxazole; CI: confidence interval; IC_50_:half maximal inhibitory concentration; pIC_50_: log (IC_50_).

**Table 2:** Average IC_50_ ± standard deviation (SD) (µg/mL and µM) and average pIC_50_ ± SD for NSC319726. Averages IC_50_ and SD were calculated on the basis of N1, N2, and N3 data of the secondary screen for all strains and tested compounds.

Strain	Average IC_50_ ± SD (µg/mL)	Average IC_50_ ± SD (µM)	Average pIC_50_ ± SD
*E. coli* ATCC 25922	0.774 ± 0.394	0.0033 ± 0.002	5.524 ± 0.245
*E. coli* ATCC 86980	0.481 ± 0.027	0.0021 ± 0.0001	5.688 ± 0.025
*E. coli* ATCC 29181	3.071 ± 0.285	0.0131 ± 0.001	4.884 ± 0.041
*E. coli* AHDRCC 81113	5.432 ± 2.731	0.0232 ± 0.012	4.668 ± 0.201

**Table 3:** Recorded MICs (μg/mL) for TMP, SMX, SXT, and NSC319726 against *E. coli* ATCC 25922 and *E. coli* AHDRCC 81113 (*n* = 2).

	Susceptibility Profile	MIC (μg/mL)			
		TMP	SMX	SXT (1:19)	NSC319726
*E. coli* ATCC 25922	S to SMX, TMP, and SXT	0.0005	64	0.12/2.4	128
		0.0005	64	0.06/1.2	256
*E. coli* AHDRCC 81113	R to SMX, TMP, and SXT	1024	>2048	>32/608	128
		1024	>2048	>32/608	128

MIC: minimum inhibitory concentration; R: resistant; S: susceptible; SMX: sulfamethoxazole; TMP: trimethoprim.

## Supplementary Materials

The following are available online at http://www.mdpi.com/2218-273X/8/4/166/s1, Figure S1: Bland–Altman plots of pairwise comparison of the potencies of the positive compounds determined in different assay runs (N1, N2, and N3) of the secondary screen in each of the tested strains (*E. coli* ATCC 25922, *E. coli* ATCC 86980, *E. coli* ATCC 29181, and *E. coli* AHDRCC 81113, respectively.
