# Supplementary material for: High-Throughput Screen Identifying the Thiosemicarbazone NSC319726 Compound as a Potent Antimicrobial Lead Against Resistant Strains of Escherichia coli
Source: Biomolecules. 2018 Dec 7;8(4):166. doi: 10.3390/biom8040166 (PMC6315430; doi:10.3390/biom8040166)
Supplement: Supplementary file 1 [file biomolecules-08-00166-s001.pdf]

Supplemental data

***E. coli* ATCC 25922**

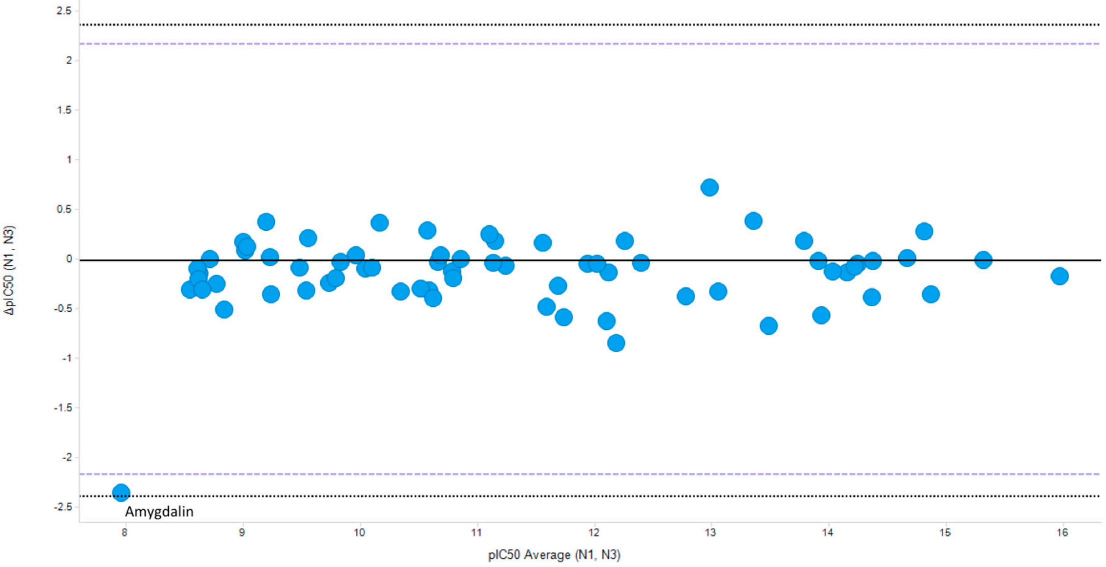

Figure S1. Bland–Altman plots of pairwise comparison of the potencies of the positive compounds determined in different assay runs (N1, N2, and N3) of the secondary screen in each of the tested strains (*E. coli* ATCC 25922, *E. coli* ATCC 86980, *E. coli* ATCC 29181, and *E. coli* AHDRCC 81113, respectively.

The continuous reference line in each of the Bland–Altman plots represents average pIC_{50} bias. Black dotted reference lines represent the 95% confidence interval of the LoA, while shaded purple dotted reference lines indicate the MSD bias interval (MSD = 2*SD of pIC_{50} differences). Positives marked with numbers outside the 95% confidence interval of the LoA and/or ±MSD interval constitute nuisance hits.

LoA: limit of agreement; MSD: minimum significant difference; SD: standard deviation.

E. coli **ATCC 25922**

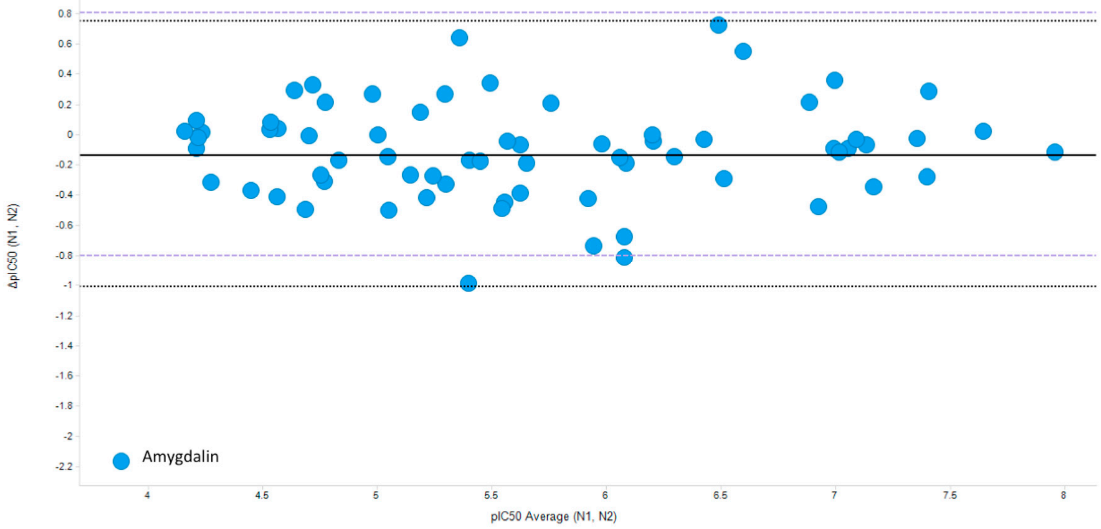

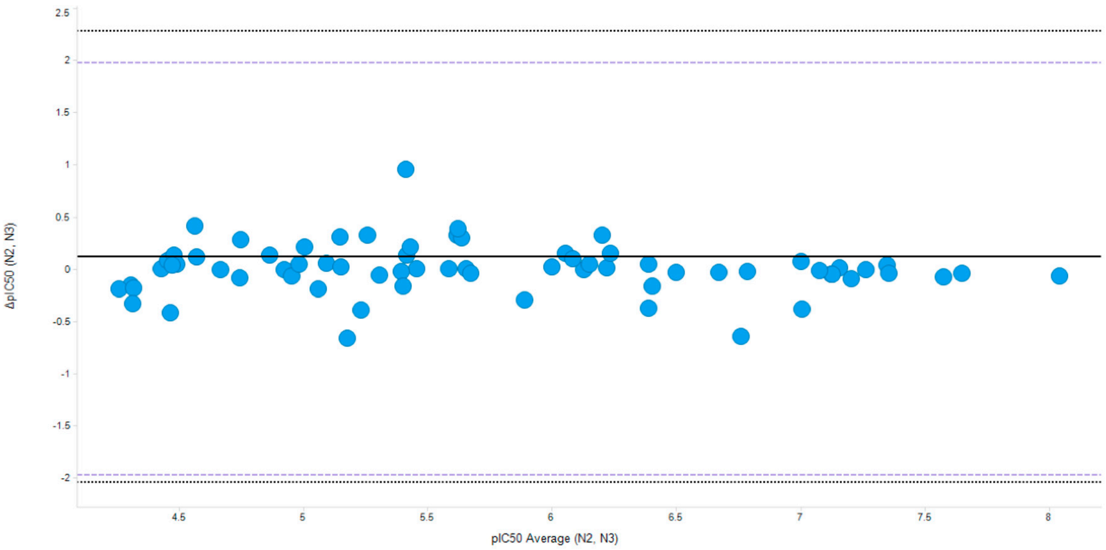

Figure S1. Continued.

E. coli **ATCC 86980**

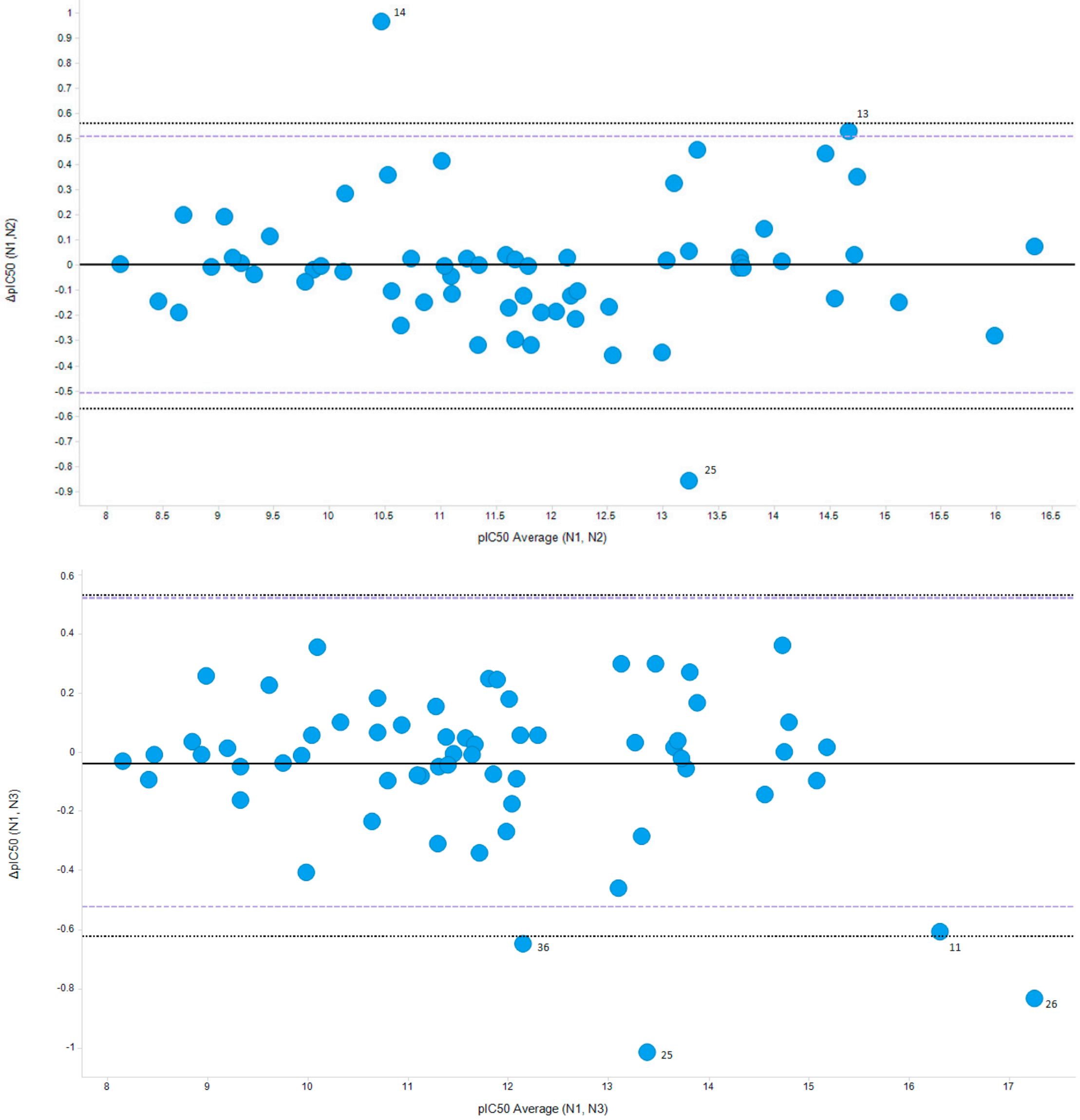

Figure S1. Continued.

***E. coli* ATCC 86980**

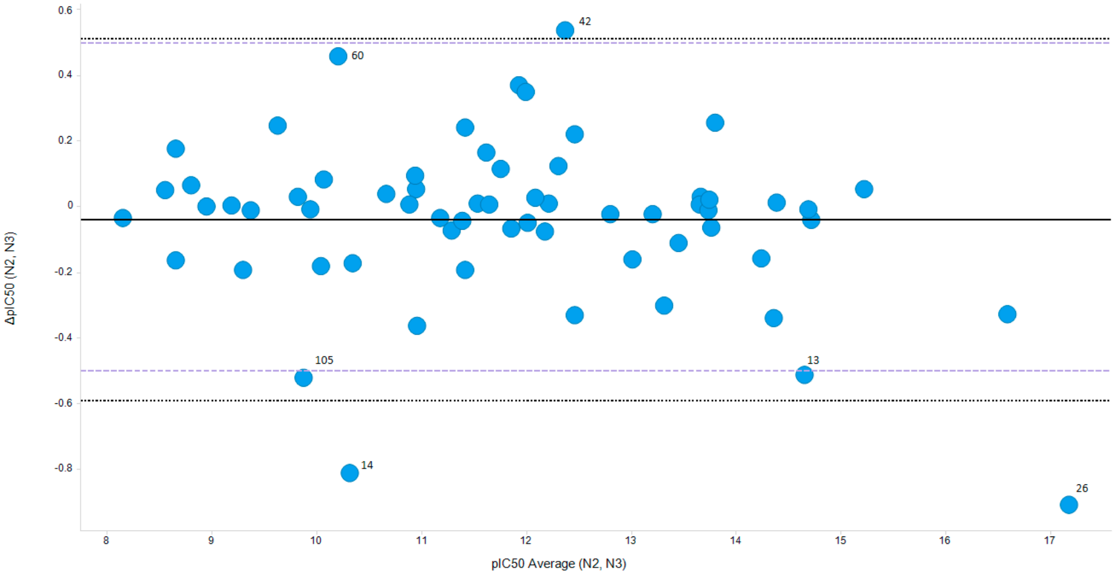

Figure S1. Continued.

***E. coli* ATCC 29181**

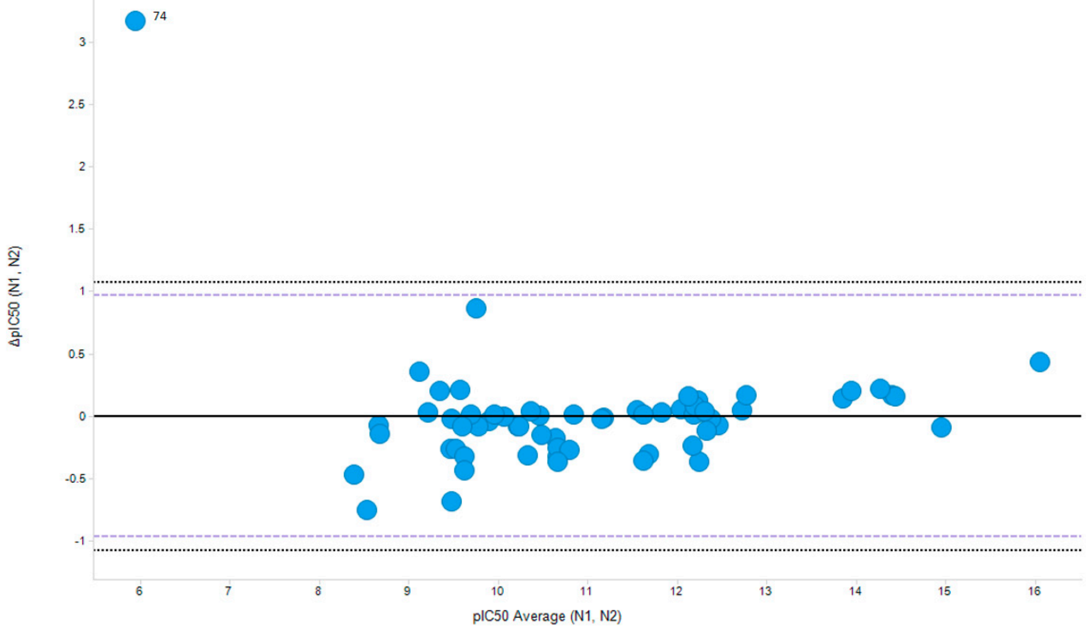

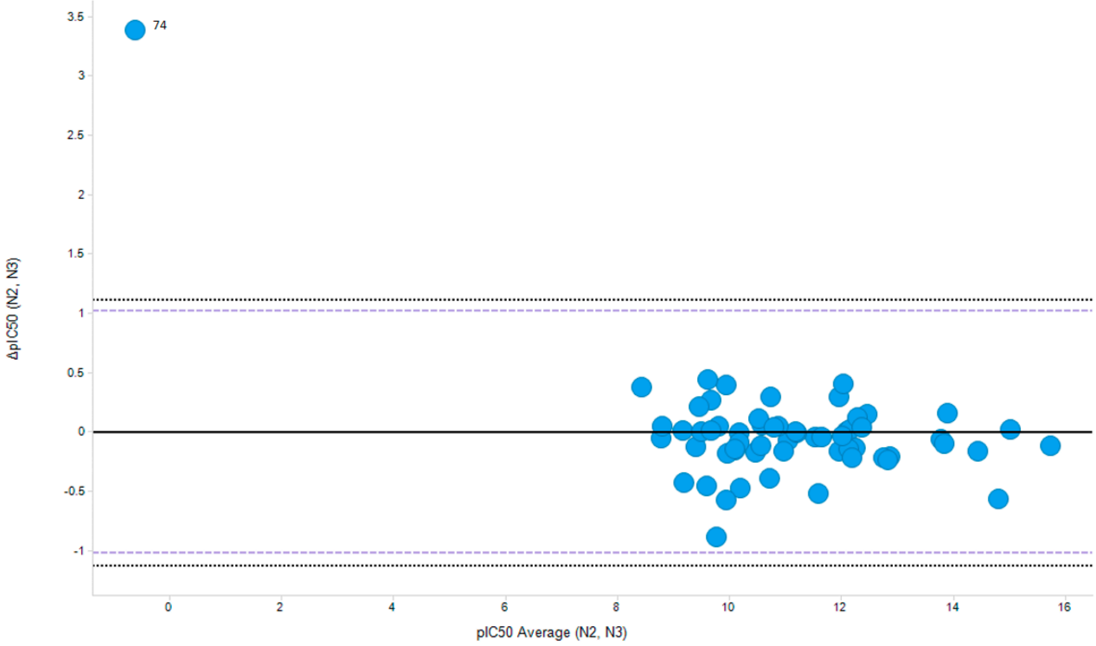

Figure S1. Continued.

***E. coli* ATCC 29181**

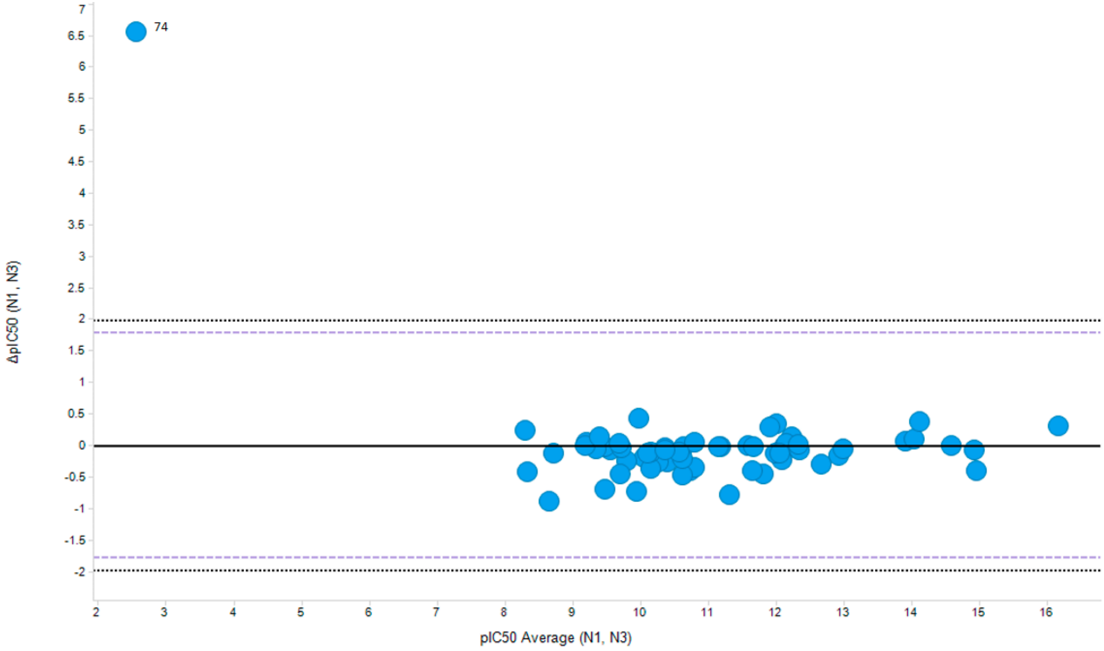

Figure S1. Continued.

E. coli **AHDRCC 81113**

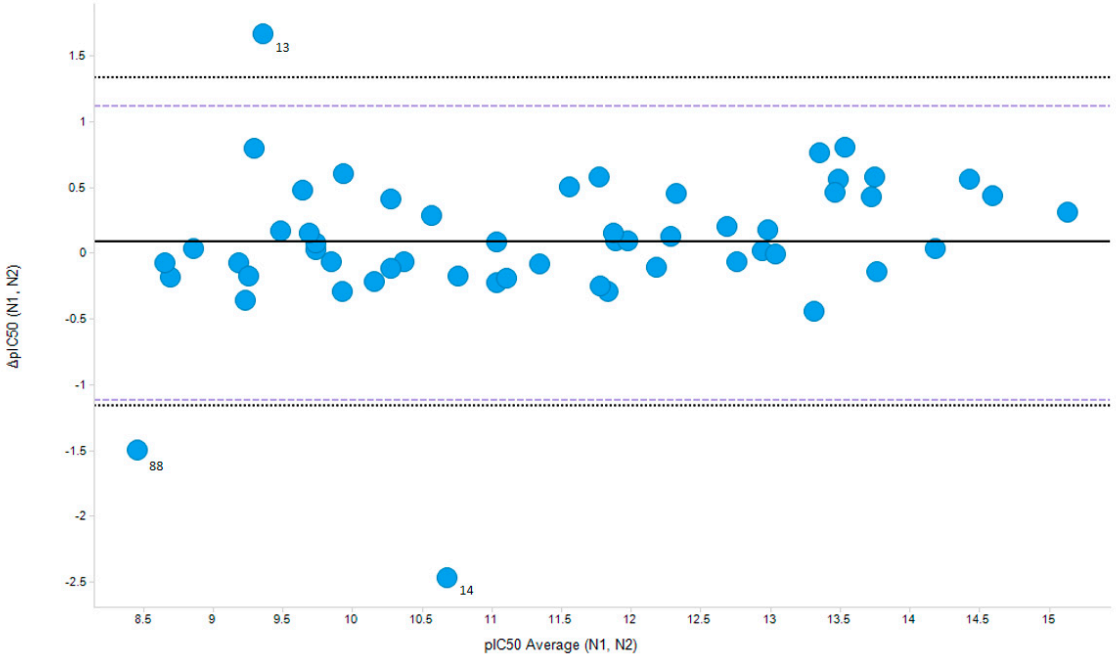

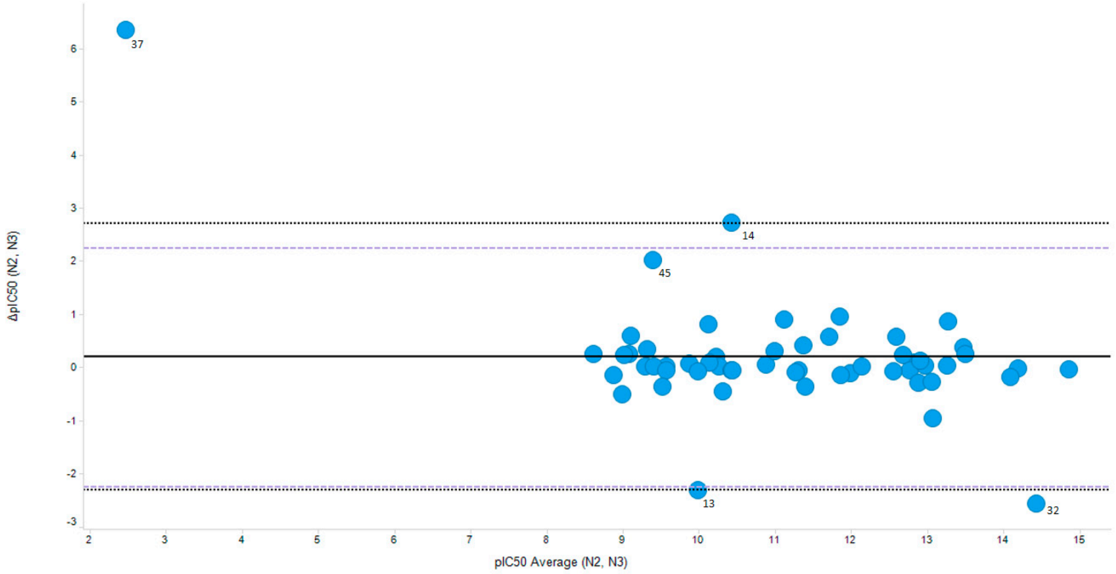

Figure S1. Continued.

***E. coli* AHDRCC 81113**

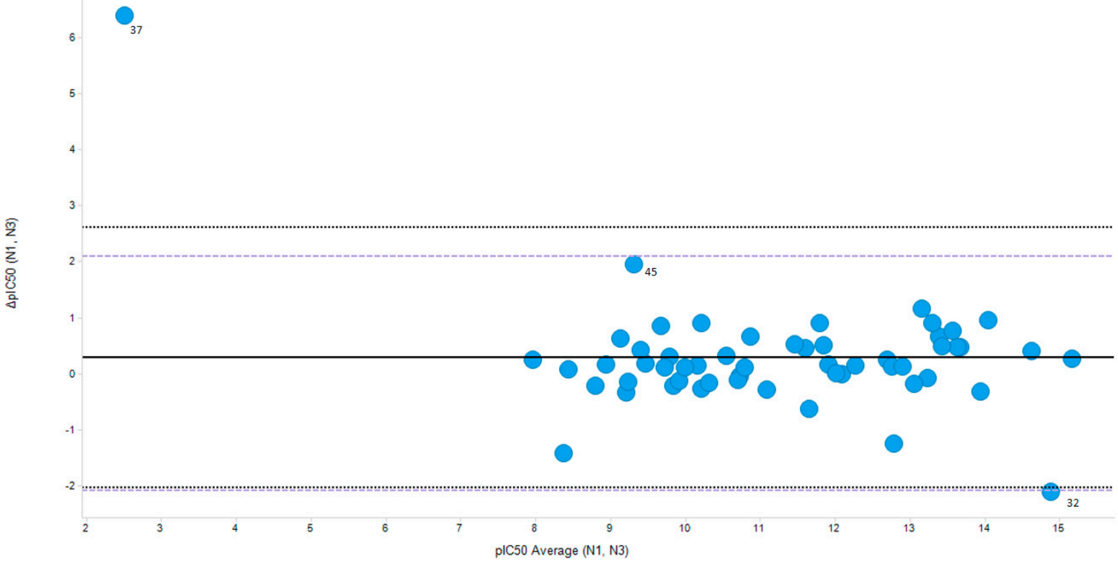

Figure S1. Continued.
